# Systemic Analyses of the Expression of TPI1 and Its Associations with Tumor Microenvironment in Lung Adenocarcinoma and Squamous Cell Carcinoma

**DOI:** 10.1155/2022/6258268

**Published:** 2022-01-25

**Authors:** Xiaodong Yang, Cong Ye, Hui Zheng, Chenyang Dai, Yuming Zhu

**Affiliations:** Department of Thoracic Surgery, Shanghai Pulmonary Hospital, Tongji University, Shanghai, China

## Abstract

**Background:**

Recent studies have shown that the expression level of triosephosphate isomerase 1 (*TPI1*) may be associated with the occurrence and metastasis of tumors, but the expression level of *TPI1* and its effect on lung adenocarcinoma (LUAD) and squamous cell carcinoma (LUSC) are not yet clear.

**Methods:**

We comprehensively explored and validated the *TPI1* expression in lung adenocarcinoma and lung squamous cell carcinoma in public datasets. The associations of *TPI1* expression with clinicopathological characteristics and prognosis were also studied in both histological types. Moreover, we analyzed the potential relations of *TPI1* with immunomodulators and immune cell infiltrations in the tumor microenvironment based on previous literatures and bioinformatic tools.

**Results:**

We found that *TPI1* was significantly overexpressed in LUAD and LUSC. Significant associations of *TPI1* expression were observed regarding age, gender, and pathological stages in LUAD. However, similar trend was only found with respect to age in LUSC. The high expression of *TPI1* was significantly associated with worse survival in LUAD, but not in LUSC. Furthermore, we explored the potential distribution and changes of *TPI1* expression in tumor microenvironment. Pathway enrichment analyses were performed to identify possible roles of *TPI1* in both lung cancers.

**Conclusions:**

*TPI1* was overexpressed in both LUAD and LUSC. Increased *TPI1* expression was correlated with poor prognosis in LUAD and changed immune cell infiltrating in various degrees in both histological types. Our study provides insights in understanding the potential roles of *TPI1* in tumor progression and immune microenvironment.

## 1. Introduction

Lung cancer is one of the most commonly diagnosed cancers, with over 1,700,000 new cases every year [1, 2]. The current histopathological classification revealed that lung adenocarcinoma (LUAD) and lung squamous cell carcinoma (LUSC) comprise majority of all lung cancers. Cancer metabolism has become the focus in cancer research and clinical oncology, including LUAD and LUSC [3]. Tumor cells are well documented to reprogram their metabolism process to support abnormal proliferation and survival in harsh conditions by mutations in oncogenes and inactivation of tumor suppressor genes [4].

Recent studies have shown that the expression level of triosephosphate isomerase 1 (*TPI1*) may be related to tumorigenesis and metastasis, but the expression level of *TPI1* and its effect on tumors are not clear yet. *TPI1* is located in the cytoplasmic and extracellular regions, which is associated with triosephosphate isomerase deficiency and giardiasis. Previous literature revealed that TP1 is significantly upregulated in intrahepatic cholangiocarcinoma and correlated with high recurrence rate [5]. Kim et al. found that *TP1* may serve as a biomarker for the diagnosis of liver metastasis in colon cancer [6]. Jiang et al. developed a prognostic model for Ewing's sarcoma which comprised *TPI1* [7]. It was also reported that *TPI1* expression was greatly decreased in hepatocellular carcinoma [8]. However, the expression changes and underlying roles of *TPI1* in LUAD and LUSC remain unknown.

Here, we comprehensively explored and validated the *TPI1* expression in LUAD and LUSC using public databases, including The Cancer Genome Atlas (TCGA) and the Gene Expression Omnibus (GEO) datasets. The associations of *TPI1* expression with clinicopathological characteristics and prognosis were also studied in both histological types. Moreover, we analyzed the potential relations of TPI1 with immune cell infiltrations in the tumor microenvironment based on previous literatures and bioinformatic tools. Our study provides insights in understanding the potential roles of *TPI1* in tumor progression and immune microenvironment, which lay the foundation for future clinical research.

## 2. Methods

### 2.1. Study Cohort and Data Processing

Level 3 RNA sequencing data of LUAD and LUSC samples were downloaded from TCGA (https://portal.gdc.cancer.gov) before January 27, 2021. We obtained 1122 samples (572 samples of LUAD dataset and 550 samples of LUSC dataset) in total. Baseline clinicopathological factors, treatment, and prognostic information were also downloaded from TCGA.

RNA sequencing data of common lung cancer cell lines (LUAD, LUSC, and small-cell lung cancer) were downloaded from the Cancer Cell Line Encyclopedia (CCLE, https://sites.broadinstitute.org/ccle) [9, 10]. We obtained 154 samples (77 samples of LUAD, 26 samples of LUSC, and 51 samples of small-cell lung cancer) in total.

We adopted the public datasets from GEO (https://www.ncbi.nlm.nih.gov/geo) as the validation cohort. We enrolled GSE30219, GSE50081, and GSE37745 which were all based on the GPL570 genechip for the comparison of *TPI1* expression among LUAD, LUSC, small-cell lung cancer, and normal lung tissue. We used a robust multichip average method by RMAExpress for background adjustment, quantile normalization, and summary to process the gene profiles [11–13]. GSE68465 and GSE157011 datasets were used for the validations of clinical and prognostic values in LUAD and LUSC, respectively. Normalized data were downloaded directly from the GEO database.

The associations of tumor microenvironment with TPI1 expression level were firstly evaluated according to several previous studies. Saltz et al. proposed a leukocyte fraction by estimating tumor-infiltrating leukocytes on hematoxylin and eosin stained slides using deep learning techniques [14]. We also used the “Estimation of STromal and Immune cells in MAlignant Tumours using Expression data (ESTIMATE)” method for the assessment of tumor microenvironment. Moreover, the CIBERSORT method was used to quantify the proportions of the immune cell in both TCGA LUAD and LUSC cohorts [15]. The CIBERSORT is an analytical tool to impute gene expression profiles and provide an estimation of the abundances of member cell types in a mixed cell population. Such mixtures could derive from both patients' solid tissues and blood profiled by array or RNA sequencing [16]. The 22 immune cells are mainly composed of B cells, T cells, macrophages, dendritic cells, plasma cells, natural killer cells, and mast cells. Second, we obtain the list of immunomodulators based on TISIDB (http://cis.hku.hk/TISIDB/). TISIDB is a web portal for tumor and immune system interaction, which integrates multiple heterogeneous data types [17]. We studied the potential associations of *TPI1* expression with immunomodulators and chemokines in TCGA LUAD and LUSC cohorts. Furthermore, we adopted Tumor Immune Single-Cell Hub (TISCH, https://tisch.comp-genomics.org/) to further explore the expression level of *TPI1* in tumor immune microenvironment. TISCH is a large-scale curated database that integrates single-cell transcriptomic profiles of 2,045,746 cells from 76 high-quality tumor datasets across 28 cancer types [18].

We performed Gene Set Enrichment Analysis (GSEA) to explore the potential effect of *TPI1* expression on LUAD and LUSC. The TCGA datasets were divided into two groups (high and low groups) stratified by *TPI1* expression level, and the enrichment of Hallmark and Kyoto Encyclopedia of Genes and Genomes (KEGG) gene sets was analyzed by GSEA, respectively. Normalized enrichment score > 1, nominal *P* value < 0.05, and false discovery rate *Q* value < 0.25 were used as screening thresholds for GSEA.

### 2.2. Statistical Analysis

All statistical analyses and graphic drawing in this study were performed by R software (version 4.0.3, R Foundation for Statistical Computing, Vienna, Austria), GraphPad Prism 8 (GraphPad Software, San Diego, CA, USA), and IBM SPSS Statistics 23.0 (IBM, Inc., Armonk, NY, USA). In each part of the study, patients were divided into high and low expression groups by the median expression level of the cohort. We adopted the Student *t*-test to compare the expression of *TPI1* between different groups. Baseline characteristics were compared by the chi-square test. Survival curves were estimated using the Kaplan-Meier method, and the log-rank test was used for comparing survival curves. Comparisons of immunological features and immune cell fractions were performed using the Mann-Whitney *U* test. In this study, a two-tailed *P* value of <0.05 was considered statistically significant.

## 3. Results

**Figure 1:**
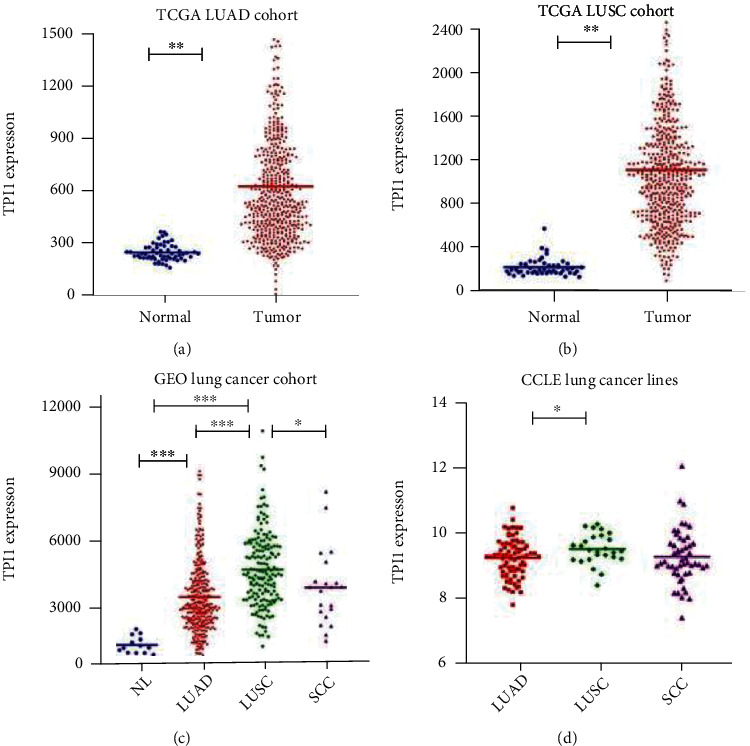
Comparison of the *TPI1* expression. (a) *TPI1* was significantly upregulated in LUAD compared with normal lung samples in TCGA (*P* < 0.001). (b) *TPI1* was significantly upregulated in LUSC compared with normal lung samples in TCGA (*P* < 0.001). (c) *TPI1* expression levels in normal lung samples, LUAD, LUSC, and small-cell lung cancer in selected GEO datasets (LUAD vs. normal sample, *P* < 0.001; LUSC vs. normal sample, *P* < 0.001; and LUSC vs. LUAD, *P* < 0.001; LUSC vs. small-cell lung cancer sample, *P* = 0.017). (d) *TPI1* expression levels in LUAD cell lines, LUSC cell lines, and small-cell lung cancer cell lines in the CCLE database (LUSC cell lines vs. LUAD cell lines, *P* = 0.032 and LUSC cell lines vs. small-cell lung cancer cell lines, *P* = 0.050).

Based on TCGA database, we obtained 572 samples (519 tumor samples and 53 lung samples) from patients with LUAD and 550 samples (501 tumor samples and 49 lung samples) from patients with LUSC. The expression level of *TPI1* was explored in both LUAD and LUSC. The results showed that *TPI1* was significantly upregulated in both LUAD and LUSC compared with normal lung tissue (*P* < 0.001 and *P* < 0.001, Figures 1(a) and 1(b)). Similar results of *TPI1* overexpression were found in the combined GEO dataset (*P* < 0.001 and *P* < 0.001, Figure 1(c)). Furthermore, we compared *TPI1* expression among common histological types of lung cancer. The *TPI1* expression of LUSC was significantly higher than that in LUAD and small-cell lung cancer (*P* < 0.001 and *P* = 0.017, Figure 1(c)). The relatively high *TPI1* expression of LUSC was also confirmed using common lung cancer cell lines in CCLE (*P* = 0.032 and *P* = 0.050, Figure 1(d)).

**Figure 2:**
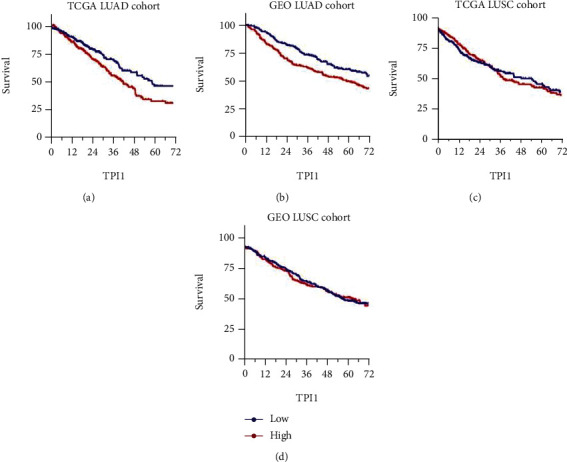
Kaplan-Meier survival curves comparing the high and low expression of *TPI1* in lung adenocarcinoma (LUAD) and squamous cell carcinoma (LUSC). (a) Survival curves comparing the *TPI1* expression high and low groups in TCGA LUAD cohort (*P* = 0.006). (b) Survival curves comparing the *TPI1* expression high and low groups in GEO LUAD cohort (*P* = 0.055). (c) Survival curves comparing the *TPI1* expression high and low groups in TCGA LUSC cohort (*P* = 0.963). (d) Survival curves comparing the *TPI1* expression high and low groups in GEO LUSC cohort (*P* = 0.571).

Next, patients with missing clinicopathological information were excluded from further analyses. All patients were divided into high and low expression groups by the median expression level in TCGA LUAD and LUSC cohorts, respectively. We assessed the potential associations of the *TPI1* expression with patients' clinicopathological factors, such as age, gender, tumor stage, and smoking history (Table 1). In TCGA LUAD cohort, we found that patients of *TPI1* low expression group tended to be older (*P* = 0.045) and consisted of more female patients (*P* = 0.021). Higher expression of *TPI1* was associated with more advanced pathological stage in LUAD (*P* < 0.001). There was no statistical difference regarding to patients' smoking history stratified by *TPI1* expression (*P* = 0.934). In TCGA LUSC cohort, similar trend of the association between age and *TPI1* expression was also observed (*P* = 0.038). No significant difference was found with respect to the distribution of patients' gender (*P* = 0.098). Meanwhile, *TPI1* expression did not correlate with the pathological stage of LUSC (*P* = 0.680) and patients' smoking history (*P* = 0.542). The prognostic values of *TPI1* in LUAD and LUSC were also evaluated. We found that high expression of *TPI1* had adverse effect on patients' survival in TCGA LUAD cohort (*P* = 0.006, Figure 2(a)). In the GEO LUAD (GSE68465) cohort, we observed that higher expression of *TPI1* was associated with worse prognosis, although the difference was not statistically significant (*P* = 0.055, Figure 2(b)). In TCGA LUSC cohort, we found that there was no significant prognostic difference in patients with LUSC stratified by the expression of *TPI1* (*P* = 0.963, Figure 2(c)). Similar result was observed in the GEO LUSC (GSE157011) cohort (*P* = 0.571, Figure 2(d)).

**Figure 3:**
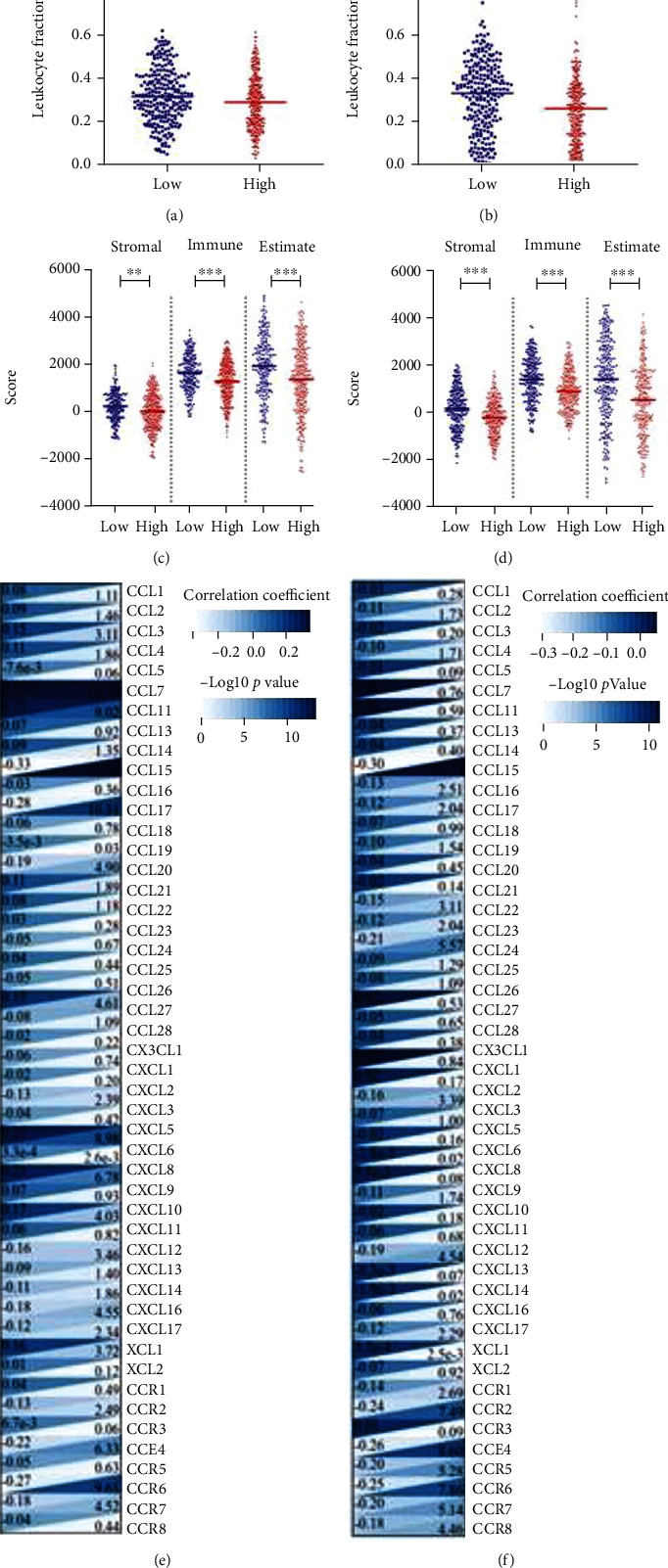
(a) Comparison of leukocyte fraction in TCGA LUAD cohort stratified by the expression of *TPI1* (*P* = 0.018). (b) Comparison of leukocyte fraction in TCGA LUSC cohort stratified by the expression of *TPI1* (*P* < 0.001). (c) Comparison of stromal, immune, and ESTIMATE scores in TCGA LUAD cohort stratified by the expression of *TPI1* (*P* = 0.001, *P* < 0.001, and *P* < 0.001). (d) Comparison of stromal, immune, and ESTIMATE scores in TCGA LUSC cohort stratified by the expression of *TPI1* (*P* < 0.001, *P* < 0.001, and *P* < 0.001). (e) Heatmap of associations of *TPI1* expression with immunomodulators and chemokines in TCGA LUAD cohort based on the TISIDB database. (f) Heatmap of associations of *TPI1* expression with immunomodulators and chemokines in TCGA LUSC cohort based on the TISIDB database.

**Figure 4:**
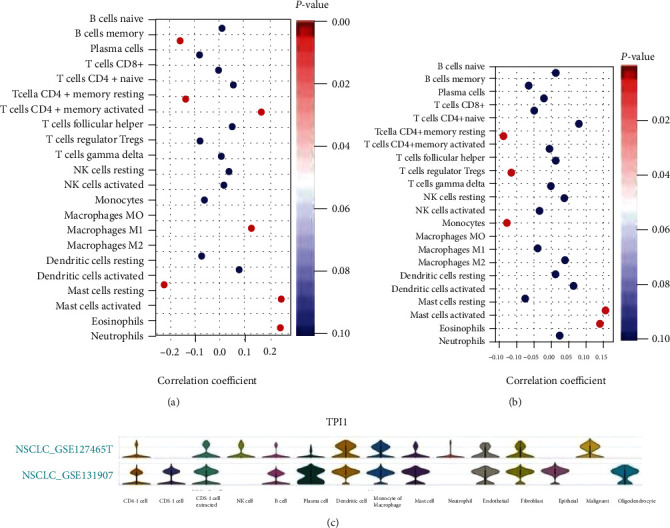
(a) Bubble plot of associations of *TPI1* expression with immune cell infiltrating level (CIBERSORT method) in TCGA LUAD cohort. (b) Bubble plot of associations of *TPI1* expression with immune cell infiltrating level (CIBERSORT method) in TCGA LUSC cohort. (c) Violin plot displays the distribution of *TPI1* expression in different cells of tumor microenvironment in GSE131907 and GSE127465 based on the TISCH database.

The tumor-infiltrating lymphocyte fractions were compared according to Saltz et al. stratified by the *TPI1* expression [14]. In both TCGA LUAD and LUSC cohorts, we found that higher expression level of *TPI1* were associated with significantly lower lymphocyte fractions (*P* = 0.018 and *P* < 0.001, Figures 3(a)–3(b)). Then, we adopted ESTIMATE method for the evaluations of tumor microenvironment. We observed that lower expression of *TPI1* was related to higher scores in patients with LUAD and LUSC (Figures 3(c) and 3(d)). Then, we studied the potential associations of *TPI1* expression with immunomodulators in TCGA LUAD and LUSC cohorts based on the TISIDB database. Significant relations were observed with chemokine, receptor, major histocompatibility complex (MHC), immunoinhibitor, and immunostimulator in both TCGA LUAD and LUSC cohorts (Figures 3(e) and 3(f) and Supplement Table 1), which suggests important roles in both metabolic and immune pathways in LUAD and LUSC. Next, we explored the potential associations of *TPI1* expression with 22 immune cell infiltrating levels by the CIBERSORT method in TCGA LUAD and LUSC cohorts. We found that *TPI1* expression was significantly associated with subclusters of B cell, T cell CD4+, macrophage, mast cell, eosinophil, and neutrophil in LUAD cohort (Figure 4(a) and Supplement Table 2). However, there were potential relations between *TPI1* expression and subclusters of T cell CD4+, T cell regulatory, monocyte, macrophage, mast cell, and eosinophil in LUSC cohort (Figure 4(b) and Supplement Table 2). In the TISCH database, we selected two lung cancer cohorts (GSE131907 and GSE127465). GSE131907 was composed of 44 patients with LUAD, while GSE127465 consists of both LUAD and LUSC patients. We studied the expression of *TPI1* at the single-cell level. The distributions of *TPI1* expression in the above datasets are displayed in Figure 4(c) and Supplement Figure 1. In GSE127465 cohort, *TPI1* was mainly expressed in dendritic cell, macrophage, and tumor cell. Similar results were observed in GSE131907 cohort, which indicated similar distribution of *TPI1* expression in LUAD and LUSC. We performed GSEA in TCGA LUAD and LUSC cohorts stratified by the expression of *TPI1*. In both LUAD and LUSC cohorts, higher *TPI1* expression was related to the enrichment of metabolic pathways and cell cycle process (Supplement Figure 2A-F). However, we noticed that higher *TPI1* expression was also associated with the enrichment of oxidative phosphorylation pathway, hypoxia-related pathway, and *P53* signaling pathway (Supplement Figure 2G-I).

## 4. Discussion

Recently, cancer metabolism has become the focus of medical research and the development of potential cancer treatment. More and more evidence indicate that metabolic changes provide cancer cells with growth advantages, especially alterations in glucose metabolism [19]. Previous studies showed that *TPI1* expression may be related to the occurrence and metastasis of tumors, but the expression level of *TPI1* and its effect on tumors are not clear yet. *TPI1*, a key enzyme in the process of carbohydrate metabolism, catalyzes the interconversion of dihydroxyacetone phosphate and D-glyceraldehyde-3-phosphate [20]. Yoshida et al. observed that *TPI1* was significantly upregulated in metastatic tumors than in primary ovarian cancer [21]. Yu et al. found that higher *TPI1* expression may be associated with a higher recurrence rate in intrahepatic cholangiocarcinoma [5]. Jiang et al. reported that *TPI1* expression was greatly decreased in hepatocellular carcinoma, which was consistent with previous study in osteosarcoma [8, 22]. It was revealed that *TPI1* expression was positively correlated with overall survival and negatively associated with tumor size and histological differentiation [8]. In this study, we adopted public datasets to explore the expression and clinical relevance of *TPI1* in LUAD and LUSC. We found that *TPI1* was significantly overexpressed in both types of lung cancers. Furthermore, *TPI1* was negatively associated with overall survival in patients with LUSC.

*TPI1* is primarily associated with triosephosphate isomerase deficiency and giardiasis [7]. *TPI1* catalyzes the stereospecific 1,2-proton shift at dihydroxyacetone phosphate to give (R)-glyceraldehyde 3-phosphate through a pair of isomeric enzyme-bound cis-enediolate phosphate intermediates [23]. The conversion of dihydroxyacetone phosphate to d-3-glyceraldehyde phosphate continues the glycolytic pathway. Therefore, *TPI1* plays an important role in the glycolysis process. Our study indicated that *TPI1* could be a predictive biomarker for LUAD and LUSC. Moreover, the metabolic changes associated with malignancy are not only in cancer cells, but also in tumor microenvironment [24]. We also explored the associations of *TPI1* with tumor microenvironment and its expression levels in various immune cells. However, it is necessary to further study the transcriptional regulation mechanism of *TPI1* and its effect in the relationship between glycolysis and immune-related pathways.

This work systematically studies the associations of *TPI1* expression with LUAD and LUSC, but there are still some shortcomings that should be mentioned. First, *TPI1* expression should be further tested in diverse lung cancer patient cohorts with different therapies. Second, the verifications of expression and the exploration of potential mechanisms require further studies *in vitro* and *in vivo*.

## 5. Conclusion

*TPI1* was significantly upregulated in LUAD and LUSC. Increased *TPI1* expression was correlated with poor prognosis in lLUAD and changed immune cell infiltrating in various degrees in both types of lung cancers. Our study provides insights in understanding the potential roles of *TPI1* in tumor progression and immune microenvironment.

## Figures and Tables

**Table 1:** Baseline clinicopathological characteristics stratified by the expression of *TPI1* in lung adenocarcinoma and lung squamous cell carcinoma.

	TCGA LUAD cohort			TCGA LUSC cohort		
	*TPI1* low	*TPI1* high	*P* value	*TPI1* low	*TPI* high	*P* value
Age^∗^	66.205 ± 9.663	64.393 ± 10.295	0.045	68.095 ± 8.272	66.481 ± 8.712	0.038
Gender			0.021			0.098
Female	151 (59.2)	125 (49.0)		71 (29.1)	55 (22.5)	
Male	104 (40.8)	130 (51.0)		173 (70.9)	189 (77.5)	
Stage^∗^			<0.001			0.680
Stage I	161 (63.4)	114 (44.7)		121 (49.6)	121 (49.6)	
Stage II	48 (18.9)	77 (30.2)		83 (34)	72 (29.5)	
Stage III	36 (14.2)	47 (18.4)		35 (14.3)	49 (20.1)	
Stage IV	9 (3.5)	17 (6.7)		5 (2)	2 (0.8)	
Smoking status^∗^			0.934			0.542
Nonsmoker	39 (15.9)	35 (14.2)		10 (4.3)	8 (3.4)	
Current smoker	46 (18.8)	73 (29.6)		61 (26)	70 (29.5)	
Reformed smoker (>15 years)	85 (34.7)	49 (19.8)		40 (17)	41 (17.3)	
Reformed smoker (≤15 years)	75 (30.6)	90 (36.4)		124 (52.8)	118 (49.8)	

^∗^Samples with missing value were excluded from the comparison in each analysis.

## Data Availability

All data could be downloaded from public databases (TCGA and GEO) and previous literatures in the reference.

## Supplementary Materials

Supplementary 1 Supplement Table 1: correlations of TPI1 expression with immune cell infiltrating levels in TCGA LUAD and LUSC cohorts.

Supplementary 2 Supplement Table 2: correlations of *TPI1* expression with chemokine, receptor, MHC, immunoinhibitor, and immunostimulator in both TCGA LUAD and LUSC cohorts based on the TISIDB database.

Supplementary 3 Supplement Figure 1: (A) single-cell cluster map of *TPI1* in GSE131907 based on the TISCH database. (B) Single-cell cluster map of *TPI1* in GSE127465 based on the TISCH database.

Supplementary 4 Supplement Figure 2: (A) GSEA showed that higher expression of *TPI1* was associated with the enrichment of glycolysis pathway (Hallmark) in TCGA LUAD cohort. (B) GSEA showed that higher expression of *TPI1* was associated with the enrichment of pyrimidine metabolism pathway (KEGG) in TCGA LUAD cohort. (C) GSEA showed that higher expression of *TPI1* was associated with the enrichment of G2M checkpoint pathway (Hallmark) in TCGA LUAD cohort. (D) GSEA showed that higher expression of *TPI1* was associated with the enrichment of citrate cycle TCA cycle pathway (KEGG) in TCGA LUSC cohort. (E) GSEA showed that higher expression of *TPI1* was associated with the enrichment of glutathione metabolism pathway (KEGG) in TCGA LUSC cohort. (F) GSEA showed that higher expression of *TPI1* was associated with the enrichment of cell cycle pathway (KEGG) in TCGA LUSC cohort. (G) GSEA showed that higher expression of *TPI1* was associated with the enrichment of oxidative phosphorylation pathway (Hallmark) in TCGA LUAD cohort. (H) GSEA showed that higher expression of *TPI1* was associated with the enrichment of hypoxia (Hallmark) in TCGA LUAD cohort. (I) GSEA showed that higher expression of *TPI1* was associated with the enrichment of P53 signaling pathway (KEGG) in TCGA LUAD cohort.
