# Supplementary material for: Systemic Analyses of the Expression of TPI1 and Its Associations with Tumor Microenvironment in Lung Adenocarcinoma and Squamous Cell Carcinoma
Source: Dis Markers. 2022 Jan 25;2022:6258268. doi: 10.1155/2022/6258268 (PMC8811541; doi:10.1155/2022/6258268)
Supplement: Supplementary 1 — Supplement Table 1: correlations of TPI1 expression with immune cell infiltrating levels in TCGA LUAD and LUSC cohorts. [file 6258268.f1.docx]

| Gene Name | Type | LUAD | | LUSC | |
| --- | --- | --- | --- | --- | --- |
|  |  | Correlation Coefficient | *P*-value | Correlation Coefficient | *P*-value |
| CCL1 | chemokine | 0.078 | 0.078 | -0.029 | 0.522 |
| CCL2 | chemokine | 0.093 | 0.035 | -0.105 | 0.019 |
| CCL3 | chemokine | 0.148 | 0.001 | -0.022 | 0.627 |
| CCL4 | chemokine | 0.109 | 0.014 | -0.104 | 0.019 |
| CCL5 | chemokine | -0.008 | 0.863 | -0.010 | 0.820 |
| CCL7 | chemokine | 0.324 | <0.001 | 0.061 | 0.172 |
| CCL8 | chemokine | 0.266 | <0.001 | 0.051 | 0.255 |
| CCL11 | chemokine | 0.069 | 0.120 | -0.036 | 0.423 |
| CCL13 | chemokine | 0.089 | 0.045 | -0.038 | 0.397 |
| CCL14 | chemokine | -0.325 | <0.001 | -0.303 | <0.001 |
| CCL15 | chemokine | -0.035 | 0.435 | -0.132 | 0.003 |
| CCL16 | chemokine | -0.285 | <0.001 | -0.116 | 0.009 |
| CCL17 | chemokine | -0.061 | 0.166 | -0.073 | 0.102 |
| CCL18 | chemokine | -0.003 | 0.937 | -0.098 | 0.029 |
| CCL19 | chemokine | -0.192 | <0.001 | -0.041 | 0.359 |
| CCL20 | chemokine | 0.110 | 0.013 | -0.016 | 0.723 |
| CCL21 | chemokine | 0.081 | 0.067 | -0.150 | 0.001 |
| CCL22 | chemokine | 0.028 | 0.530 | -0.116 | 0.009 |
| CCL23 | chemokine | -0.055 | 0.215 | -0.208 | <0.001 |
| CCL24 | chemokine | 0.040 | 0.365 | -0.087 | 0.051 |
| CCL25 | chemokine | -0.045 | 0.307 | -0.078 | 0.081 |
| CCL26 | chemokine | 0.185 | <0.001 | 0.047 | 0.294 |
| CCL27 | chemokine | -0.077 | 0.081 | -0.055 | 0.223 |
| CCL28 | chemokine | -0.023 | 0.596 | -0.036 | 0.421 |
| CX3CL1 | chemokine | -0.059 | 0.183 | 0.065 | 0.144 |
| CXCL1 | chemokine | -0.022 | 0.626 | 0.019 | 0.672 |
| CXCL2 | chemokine | -0.127 | 0.004 | -0.157 | <0.001 |
| CXCL3 | chemokine | -0.039 | 0.376 | -0.073 | 0.101 |
| CXCL5 | chemokine | 0.265 | <0.001 | -0.017 | 0.697 |
| CXCL6 | chemokine | <0.001 | 0.994 | -0.003 | 0.950 |
| CXCL8 | chemokine | 0.229 | <0.001 | 0.010 | 0.827 |
| CXCL9 | chemokine | 0.069 | 0.118 | -0.105 | 0.018 |
| CXCL10 | chemokine | 0.172 | <0.001 | -0.020 | 0.661 |
| CXCL11 | chemokine | 0.064 | 0.150 | -0.056 | 0.207 |
| CXCL12 | chemokine | -0.157 | <0.001 | -0.186 | <0.001 |
| CXCL13 | chemokine | -0.091 | 0.040 | -0.008 | 0.850 |
| CXCL14 | chemokine | -0.109 | 0.014 | -0.002 | 0.966 |
| CXCL16 | chemokine | -0.184 | <0.001 | -0.061 | 0.174 |
| CXCL17 | chemokine | -0.125 | 0.005 | -0.125 | 0.005 |
| XCL1 | chemokine | 0.164 | <0.001 | <0.001 | 0.994 |
| XCL2 | chemokine | 0.013 | 0.760 | -0.069 | 0.121 |
| CCR1 | receptor | 0.044 | 0.323 | -0.137 | 0.002 |
| CCR2 | receptor | -0.130 | 0.003 | -0.244 | <0.001 |
| CCR3 | receptor | 0.007 | 0.879 | 0.010 | 0.818 |
| CCR4 | receptor | -0.220 | <0.001 | -0.262 | <0.001 |
| CCR5 | receptor | -0.053 | 0.233 | -0.202 | <0.001 |
| CCR6 | receptor | -0.275 | <0.001 | -0.250 | <0.001 |
| CCR7 | receptor | -0.183 | <0.001 | -0.199 | <0.001 |
| CCR8 | receptor | -0.040 | 0.361 | -0.184 | <0.001 |
| CCR9 | receptor | -0.103 | 0.020 | -0.068 | 0.131 |
| CCR10 | receptor | 0.148 | 0.001 | -0.076 | 0.088 |
| CXCR1 | receptor | 0.050 | 0.263 | -0.076 | 0.090 |
| CXCR2 | receptor | 0.017 | 0.699 | -0.115 | 0.010 |
| CXCR3 | receptor | -0.050 | 0.254 | -0.202 | <0.001 |
| CXCR4 | receptor | -0.147 | 0.001 | -0.179 | <0.001 |
| CXCR5 | receptor | -0.181 | <0.001 | -0.155 | 0.001 |
| CXCR6 | receptor | -0.064 | 0.149 | -0.155 | <0.001 |
| XCR1 | receptor | -0.176 | <0.001 | -0.120 | 0.007 |
| CX3CR1 | receptor | -0.167 | <0.001 | -0.166 | <0.001 |
| B2M | MHC | 0.009 | 0.836 | -0.089 | 0.046 |
| HLA-A | MHC | 0.017 | 0.698 | -0.056 | 0.211 |
| HLA-B | MHC | -0.025 | 0.571 | -0.099 | 0.026 |
| HLA-C | MHC | 0.001 | 0.977 | -0.066 | 0.143 |
| HLA-DMA | MHC | -0.270 | <0.001 | -0.292 | <0.001 |
| HLA-DMB | MHC | -0.134 | 0.002 | -0.228 | <0.001 |
| HLA-DOA | MHC | -0.198 | <0.001 | -0.272 | <0.001 |
| HLA-DOB | MHC | -0.248 | <0.001 | -0.211 | <0.001 |
| HLA-DPA1 | MHC | -0.189 | <0.001 | -0.249 | <0.001 |
| HLA-DPB1 | MHC | -0.231 | <0.001 | -0.274 | <0.001 |
| HLA-DQA1 | MHC | -0.127 | 0.004 | -0.183 | <0.001 |
| HLA-DQA2 | MHC | -0.079 | 0.074 | -0.195 | <0.001 |
| HLA-DQB1 | MHC | -0.182 | <0.001 | -0.201 | <0.001 |
| HLA-DRA | MHC | -0.150 | 0.001 | -0.214 | <0.001 |
| HLA-DRB1 | MHC | -0.188 | <0.001 | -0.189 | <0.001 |
| HLA-E | MHC | -0.184 | <0.001 | -0.225 | <0.001 |
| HLA-F | MHC | -0.093 | 0.035 | -0.138 | 0.002 |
| HLA-G | MHC | -0.032 | 0.470 | -0.076 | 0.090 |
| TAP1 | MHC | 0.178 | <0.001 | -0.031 | 0.489 |
| TAP2 | MHC | 0.127 | 0.004 | -0.154 | 0.001 |
| TAPBP | MHC | -0.033 | 0.456 | -0.047 | 0.298 |
| ADORA2A | Immunoinhibitor | -0.147 | 0.001 | -0.206 | <0.001 |
| BTLA | Immunoinhibitor | -0.248 | <0.001 | -0.244 | <0.001 |
| CD160 | Immunoinhibitor | -0.224 | <0.001 | -0.158 | <0.001 |
| CD244 | Immunoinhibitor | -0.063 | 0.155 | -0.085 | 0.057 |
| CD274 | Immunoinhibitor | 0.209 | <0.001 | 0.067 | 0.134 |
| CD96 | Immunoinhibitor | -0.139 | 0.002 | -0.207 | <0.001 |
| CSF1R | Immunoinhibitor | -0.005 | 0.910 | -0.199 | <0.001 |
| CTLA4 | Immunoinhibitor | -0.093 | 0.036 | -0.229 | <0.001 |
| HAVCR2 | Immunoinhibitor | 0.063 | 0.157 | -0.148 | 0.001 |
| IDO1 | Immunoinhibitor | 0.034 | 0.442 | -0.055 | 0.220 |
| IL10 | Immunoinhibitor | -0.039 | 0.381 | -0.051 | 0.253 |
| IL10RB | Immunoinhibitor | 0.076 | 0.084 | -0.021 | 0.641 |
| KDR | Immunoinhibitor | -0.078 | 0.077 | -0.161 | <0.001 |
| KIR2DL1 | Immunoinhibitor | 0.066 | 0.134 | -0.036 | 0.426 |
| KIR2DL3 | Immunoinhibitor | 0.094 | 0.034 | 0.050 | 0.267 |
| LAG3 | Immunoinhibitor | 0.080 | 0.072 | -0.058 | 0.196 |
| LGALS9 | Immunoinhibitor | -0.120 | 0.006 | -0.066 | 0.139 |
| PDCD1 | Immunoinhibitor | 0.051 | 0.247 | -0.214 | <0.001 |
| PDCD1LG2 | Immunoinhibitor | 0.142 | 0.001 | 0.034 | 0.447 |
| PVRL2 | Immunoinhibitor | 0.186 | <0.001 | -0.099 | 0.027 |
| TGFB1 | Immunoinhibitor | 0.021 | 0.641 | -0.042 | 0.349 |
| TGFBR1 | Immunoinhibitor | 0.044 | 0.316 | -0.014 | 0.749 |
| TIGIT | Immunoinhibitor | -0.110 | 0.012 | -0.231 | <0.001 |
| VTCN1 | Immunoinhibitor | -0.012 | 0.787 | 0.004 | 0.922 |
| BTNL2 | Immunostimulator | -0.084 | 0.058 | -0.076 | 0.091 |
| C10orf54 | Immunostimulator | -0.162 | <0.001 | -0.312 | <0.001 |
| CD27 | Immunostimulator | -0.219 | <0.001 | -0.288 | <0.001 |
| CD276 | Immunostimulator | 0.378 | <0.001 | 0.188 | <0.001 |
| CD28 | Immunostimulator | -0.191 | <0.001 | -0.253 | <0.001 |
| CD40 | Immunostimulator | 0.039 | 0.378 | -0.037 | 0.407 |
| CD40LG | Immunostimulator | -0.325 | <0.001 | -0.260 | <0.001 |
| CD48 | Immunostimulator | -0.116 | 0.009 | -0.156 | <0.001 |
| CD70 | Immunostimulator | 0.186 | <0.001 | -0.017 | 0.712 |
| CD80 | Immunostimulator | -0.045 | 0.307 | -0.059 | 0.187 |
| CD86 | Immunostimulator | 0.043 | 0.327 | -0.145 | 0.001 |
| CXCL12 | Immunostimulator | -0.157 | <0.001 | -0.186 | <0.001 |
| CXCR4 | Immunostimulator | -0.147 | 0.001 | -0.179 | <0.001 |
| ENTPD1 | Immunostimulator | -0.172 | <0.001 | -0.237 | <0.001 |
| HHLA2 | Immunostimulator | 0.005 | 0.918 | -0.085 | 0.058 |
| ICOS | Immunostimulator | -0.067 | 0.131 | -0.196 | <0.001 |
| ICOSLG | Immunostimulator | -0.002 | 0.973 | -0.029 | 0.521 |
| IL2RA | Immunostimulator | 0.104 | 0.019 | -0.054 | 0.226 |
| IL6 | Immunostimulator | 0.068 | 0.124 | 0.015 | 0.740 |
| IL6R | Immunostimulator | -0.312 | <0.001 | -0.106 | 0.018 |
| KLRC1 | Immunostimulator | 0.178 | <0.001 | -0.084 | 0.060 |
| KLRK1 | Immunostimulator | -0.149 | 0.001 | -0.201 | <0.001 |
| LTA | Immunostimulator | -0.126 | 0.004 | -0.160 | <0.001 |
| MICB | Immunostimulator | 0.285 | <0.001 | 0.051 | 0.253 |
| NT5E | Immunostimulator | 0.239 | <0.001 | -0.006 | 0.885 |
| PVR | Immunostimulator | 0.340 | <0.001 | -0.051 | 0.256 |
| RAET1E | Immunostimulator | 0.193 | <0.001 | -0.087 | 0.051 |
| TMEM173 | Immunostimulator | -0.232 | <0.001 | -0.155 | <0.001 |
| TMIGD2 | Immunostimulator | <0.001 | 0.996 | -0.107 | 0.017 |
| TNFRSF13B | Immunostimulator | -0.276 | <0.001 | -0.277 | <0.001 |
| TNFRSF13C | Immunostimulator | -0.219 | <0.001 | -0.127 | <0.001 |
| TNFRSF14 | Immunostimulator | -0.202 | <0.001 | -0.237 | <0.001 |
| TNFRSF17 | Immunostimulator | -0.183 | <0.001 | -0.262 | <0.001 |
| TNFRSF18 | Immunostimulator | 0.106 | 0.016 | 0.074 | 0.099 |
| TNFRSF25 | Immunostimulator | -0.032 | 0.468 | -0.171 | <0.001 |
| TNFRSF4 | Immunostimulator | 0.028 | 0.533 | -0.033 | 0.457 |
| TNFRSF8 | Immunostimulator | 0.017 | 0.704 | -0.055 | 0.219 |
| TNFRSF9 | Immunostimulator | 0.103 | 0.020 | -0.051 | 0.254 |
| TNFSF13 | Immunostimulator | -0.197 | <0.001 | -0.136 | 0.002 |
| TNFSF13B | Immunostimulator | -0.028 | 0.530 | -0.144 | 0.001 |
| TNFSF14 | Immunostimulator | -0.198 | <0.001 | -0.243 | <0.001 |
| TNFSF15 | Immunostimulator | -0.208 | <0.001 | 0.076 | 0.090 |
| TNFSF18 | Immunostimulator | 0.171 | <0.001 | -0.004 | 0.923 |
| TNFSF4 | Immunostimulator | 0.209 | <0.001 | -0.047 | 0.296 |
| TNFSF9 | Immunostimulator | 0.112 | 0.011 | 0.011 | 0.798 |
| ULBP1 | Immunostimulator | 0.085 | 0.053 | 0.166 | <0.001 |

Supplement Table 2: Correlations of TPI1 expression with chemokine, receptor, MHC, immunoinhibitor and immunostimulator in both TCGA LUAD and LUSC cohorts based on TISIDB database.
