# Supplementary material for: Systemic Analyses of the Expression of TPI1 and Its Associations with Tumor Microenvironment in Lung Adenocarcinoma and Squamous Cell Carcinoma
Source: Dis Markers. 2022 Jan 25;2022:6258268. doi: 10.1155/2022/6258268 (PMC8811541; doi:10.1155/2022/6258268)
Supplement: Supplementary 2 — Supplement Table 2: correlations of TPI1 expression with chemokine, receptor, MHC, immunoinhibitor, and immunostimulator in both TCGA LUAD and LUSC cohorts based on the TISIDB database. [file 6258268.f2.docx]

|  | TCGA LUAD cohort | | TCGA LUSC cohort | |
| --- | --- | --- | --- | --- |
| Immune cell | Correlation Coefficent | *P*-value | Correlation Coefficent | *P*-value |
| B cells naive | 0.006 | 0.902 | 0.013 | 0.795 |
| B cells memory | -0.163 | 0.001 | -0.063 | 0.215 |
| Plasma cells | -0.079 | 0.113 | -0.02 | 0.698 |
| T cells CD8+ | -0.007 | 0.888 | -0.051 | 0.323 |
| T cells CD4+ naive | 0.054 | 0.278 | 0.079 | 0.121 |
| T cells CD4+ memory resting | -0.14 | 0.005 | -0.138 | 0.007 |
| T cells CD4+ memory activated | 0.163 | 0.001 | -0.005 | 0.918 |
| T cells follicular helper | 0.048 | 0.337 | 0.012 | 0.812 |
| T cells regulatory Tregs | -0.08 | 0.11 | -0.116 | 0.023 |
| T cells gamma delta | 0.007 | 0.892 | -0.001 | 0.989 |
| NK cells resting | 0.031 | 0.535 | 0.037 | 0.473 |
| NK cells activated | 0.019 | 0.711 | -0.035 | 0.497 |
| Monocytes | -0.061 | 0.227 | -0.128 | 0.012 |
| Macrophages M0 | 0.102 | 0.042 | 0.112 | 0.028 |
| Macrophages M1 | 0.128 | 0.011 | -0.039 | 0.448 |
| Macrophages M2 | 0.101 | 0.045 | 0.039 | 0.451 |
| Dendritic cells resting | -0.07 | 0.165 | 0.012 | 0.81 |
| Dendritic cells activated | 0.083 | 0.097 | 0.066 | 0.198 |
| Mast cells resting | -0.223 | 0 | -0.075 | 0.142 |
| Mast cells activated | 0.245 | 0 | 0.155 | 0.002 |
| Eosinophils | 0.103 | 0.039 | 0.14 | 0.006 |
| Neutrophils | 0.245 | 0 | 0.024 | 0.641 |

Supplement Table 1: Correlations of TPI1 expression with Immune cell infiltrating levels in TCGA LUAD and LUSC cohorts
