# Supplementary figures and images for: Systemic Analyses of the Expression of TPI1 and Its Associations with Tumor Microenvironment in Lung Adenocarcinoma and Squamous Cell Carcinoma
Source: Dis Markers. 2022 Jan 25;2022:6258268. doi: 10.1155/2022/6258268 (PMC8811541; doi:10.1155/2022/6258268)

**A**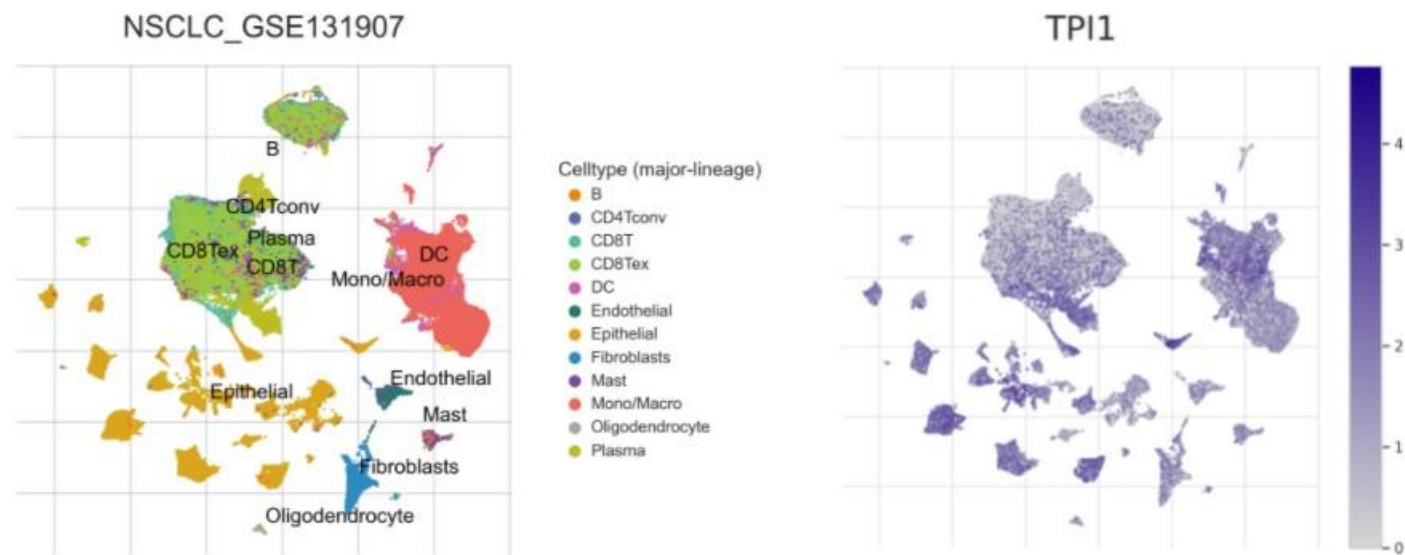**B**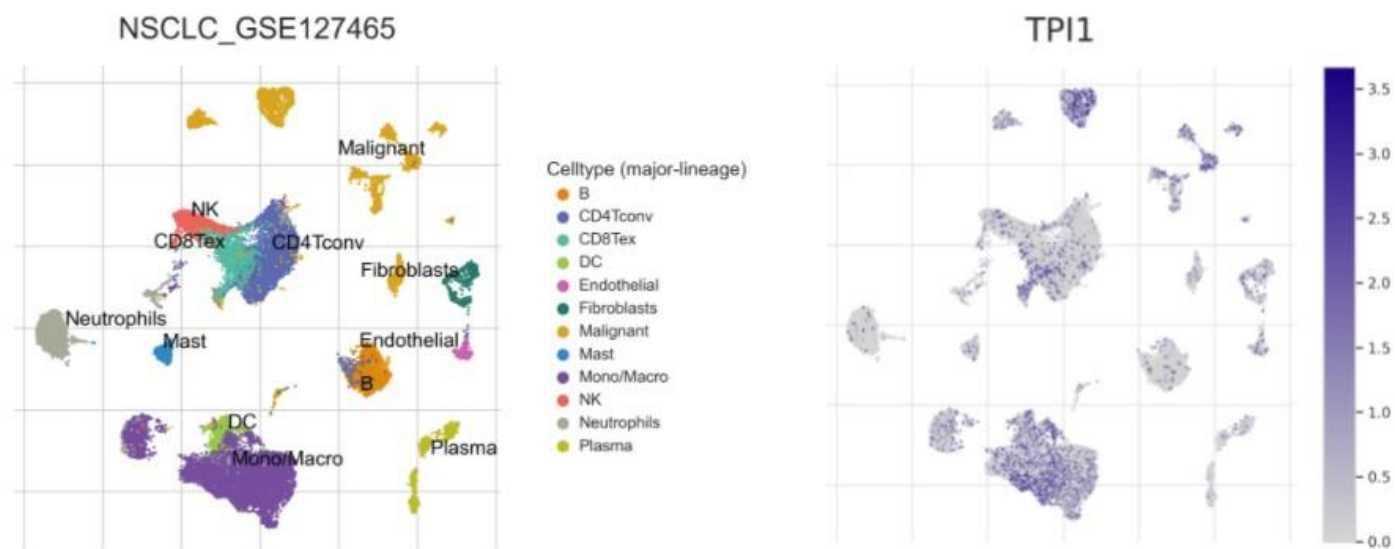

Supplement: Supplementary 3 — Supplement Figure 1: (A) single-cell cluster map of TPI1 in GSE131907 based on the TISCH database. (B) Single-cell cluster map of TPI1 in GSE127465 based on the TISCH database. [file 6258268.f3.pdf]

A

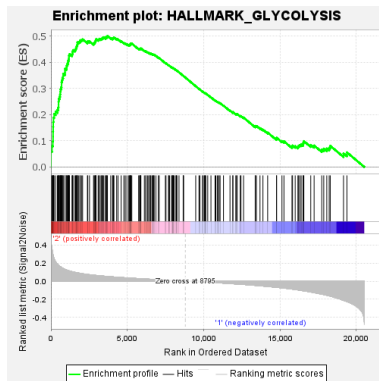

B

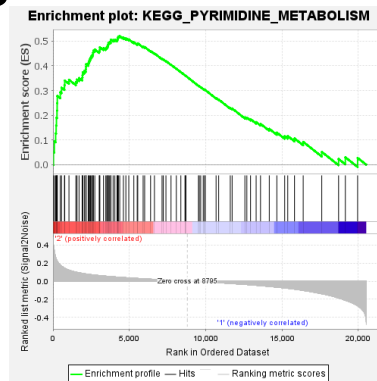

C

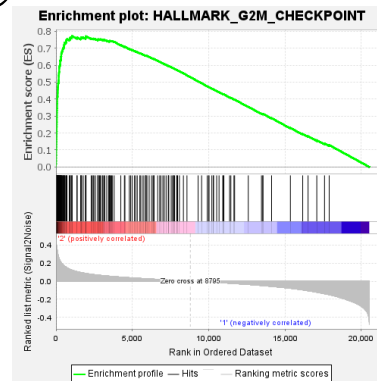

D

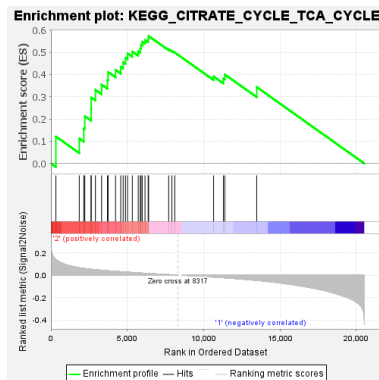

E

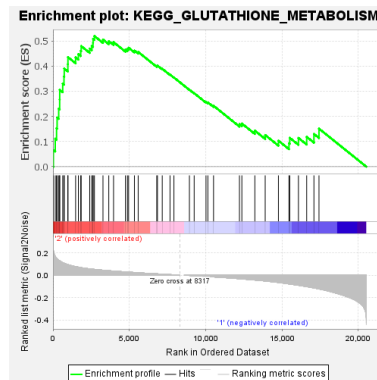

F

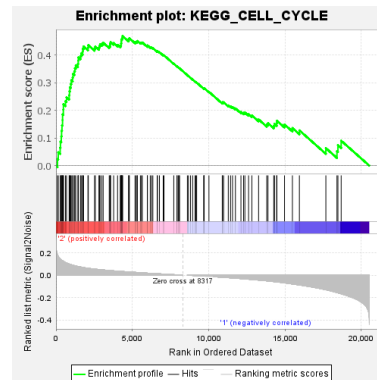

G

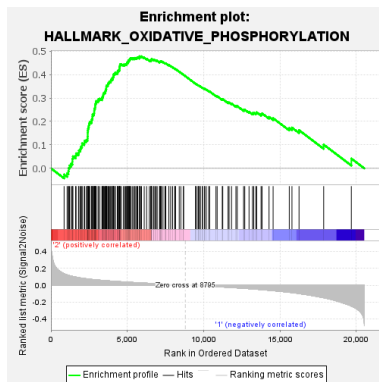

H

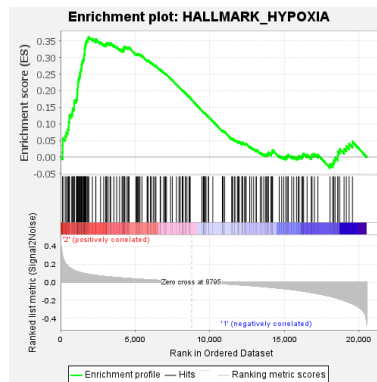

I

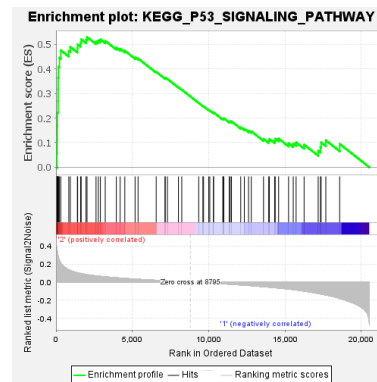

Supplement: Supplementary 4 — Supplement Figure 2: (A) GSEA showed that higher expression of TPI1 was associated with the enrichment of glycolysis pathway (Hallmark) in TCGA LUAD cohort. (B) GSEA showed that higher expression of TPI1 was associated with the enrichment of pyrimidine metabolism pathway (KEGG) in TCGA LUAD cohort. (C) GSEA showed that higher expression of TPI1 was associated with the enrichment of G2M checkpoint pathway (Hallmark) in TCGA LUAD cohort. (D) GSEA showed that higher expression of TPI1 was associated with the enrichment of citrate cycle TCA cycle pathway (KEGG) in TCGA LUSC cohort. (E) GSEA showed that higher expression of TPI1 was associated with the enrichment of glutathione metabolism pathway (KEGG) in TCGA LUSC cohort. (F) GSEA showed that higher expression of TPI1 was associated with the enrichment of cell cycle pathway (KEGG) in TCGA LUSC cohort. (G) GSEA showed that higher expression of TPI1 was associated with the enrichment of oxidative phosphorylation pathway (Hallmark) in TCGA LUAD cohort. (H) GSEA showed that higher expression of TPI1 was associated with the enrichment of hypoxia (Hallmark) in TCGA LUAD cohort. (I) GSEA showed that higher expression of TPI1 was associated with the enrichment of P53 signaling pathway (KEGG) in TCGA LUAD cohort. [file 6258268.f4.pdf]
